# Efficacy and Safety of Rimegepant for the Acute Treatment of Migraine: Evidence From Randomized Controlled Trials

**DOI:** 10.3389/fphar.2019.01577

**Published:** 2020-01-24

**Authors:** Bixi Gao, Yanbo Yang, Zilan Wang, Yue Sun, Zhouqing Chen, Yun Zhu, Zhong Wang

**Affiliations:** Department of Neurosurgery & Brain and Nerve Research Laboratory, The First Affiliated Hospital of Soochow University, Suzhou, China

**Keywords:** rimegepant, migraine, freedom from pain, freedom from most bothersome symptom, meta-analysis

## Abstract

**Background:**

As one of the novel therapeutic drugs that targets Calcitonin gene-related peptide (CGRP), 75 mg rimegepant has been used for the acute management of migraine, which is one of the most common neurological diseases worldwide. Several clinical trials have been conducted to investigate the efficacy and safety of rimegepant for the acute management of migraine, but no systematic review of existing literature has been performed. We therefore performed a meta-analysis to investigate the efficacy and safety of rimegepant in treatment of patients with migraine.

**Method:**

Pubmed, Embased, and Cochrane Library were searched from January 2001 to August 2019 for randomized controlled trials (RCTs). Four RCTs with 3,827 patients were finally included in our study.

**Result:**

We pooled 3,827 patients from four RCTs, and the primary endpoints were freedom from pain, most bothersome symptom, and pain relief at 2 hr post dose. We found that 75 mg rimegepant led to significant freedom from pain (P < 0.001), pain relief (P < 0.001), and freedom from the most bothersome symptom (P < 0.001) at 2 hr post dose compared with the placebo. In addition, there was no statistically significant increase in adverse events compared with the placebo.

**Conclusions:**

75 mg rimegepant had good efficacy and safety for acute treatment of migraine. Further studies are needed to compare the efficacy of rimegepant with traditional drugs for acute management of migraine.

## Introduction

Migraine is one of the most common neurological diseases, and it affects more than 16% of people in Western countries. (Lipton et al., 2007) Many drugs are currently available for the acute treatment of migraine, such as triptans, non-steroidal anti-inflammatory drugs (NSAIDs), acetaminophen, ergots, and opioids. However, these drugs either have more contraindications or adverse effects. In past decades, serotonin 5-HT_1B_ and 5-HT_1D_ receptor agonists (triptans) were most widely used for acute treatment of migraine. However, only 34% patients who use triptans s a good response while 30–40% patients have recurrences of pain. (Perry and Markham, 1998; Geraud et al., 2003; Cortelli et al., 2011; Lipton et al., 2017a) In addition, more than 50% patients who received triptans developed mild and severe adverse effects, and among these were cardiovascular effects, which are related to the vasoconstriction effect and this can cause fatal consequences and limit the use of triptans in patients with cardiovascular disease comorbidity. (Derry et al., 2012) Many patients discounted these drugs due to the lack of efficacy and/or bothersome adverse effects. (Corrigendum, 2016; Lipton et al., 2017b) Thus, there is still an urgent need for the development of new therapeutic methods for acute management of migraine headache.

CGRP has been found to be closely related to the pathogenesis of migraine. It has been observed that increased CGRP serum concentrations are correlated with migraine attack. (Edvinsson, 2019) Several clinical trials have shown that CGRP receptor antagonists have significant relieving effects on symptoms of migraine. (Villalon and Olesen, 2009; Ho et al., 2010) The possible mechanism of action includes causing central neurogenic vasodilation, inhibiting both vascular nociceptive transmission, and thalamic trigeminal nociceptive activation. (Storer et al., 2004; Summ et al., 2010) There are several CGRP receptor antagonists (called gepants), including olcegepant (BIBN4096BS), telcagepant (MK-0974), (MK-3207), (BI-44370 TA), rimegepant (BMS-927711), and ubrogepant (MK-1602) (Negro and Martelletti, 2019; Xu and Sun, 2019). According to previous studies (Xu and Sun, 2019), olcegepant and BI-44370 have good efficacy against migraine but come with relatively high toxicity. These two types of gepants thus have limited clinical usefulness. Rimegepant is therefore safer and has good efficacy, deserving more research.

Rimegepant is a small molecule CGRP receptor antagonists. For acute management of migraine, rapid pain relief is deemed to be a major concern, and freedom from pain at 2 hr post dose is therefore commonly used as a primary criterion for evaluation of these drugs. Nausea, dizziness, urinary tract infection, and liver injury are the most commonly reported adverse events in patients treated with rimegepant. Marcus’s study has identified the optimal dose of the rimegepant used in the migraine as 75 mg, as it was found that this dose ensured the same clinical efficacy and fewer adverse events than higher doses. (Marcus et al., 2014) The most recent studies thus selected 75 mg rimegepant for the acute pain management of migraine. Recent studies have inconsistent results regarding the efficacy and tolerability of rimegepant.(Loder and Tfelt-Hansen, 2019); Our meta-analysis included four RCTs c (Marcus et al., 2014; 60th Annual Scientific Meeting American Headache Society, 2018; Lipton et al., 2019; Croop et al., 2019).

## Methods

### Search Strategy

A search was made for several terms in Pubmed, Embased, and Cochrane Library—[(“rimegepant, migraine”), (“BMS-927711 and migraine”), (“BHV-3000 and migraine”)]—until August 2019 to find potentially eligible studies. In addition, we manually screened reference lists from RCTs and systematic reviews to ensure all relevant studies had been included in this study.

### Inclusion and Exclusion Criteria

Inclusion criteria were as follows: (a) study type: RCTs; (b) language restriction: no language restriction was applied in our study; (c) participants: patients aged >18 years with migraine for at least 1 year; (d) intervention: rimegepant and placebo; (e) outcomes: efficacy outcomes including freedom from pain, freedom from most bothersome symptom and pain relief at 2 hr, and safety outcomes. Exclusion criteria were as follows: (a) study types: case reports, case reviews, post-hoc analyses studies, retrospective studies, and cohort studies; (b) patients with a history of any clinically significant or unstable medical condition; and patients who received nonbiologic investigational agents within 30 days of the baseline visit or received biologic investigational agents within 90 days before the baseline visit.

### Study Selection and Data Collection

All articles and reference lists of the RCTs and reviews from the systematic search in the electronic database were assessed in accordance with the mentioned inclusion and exclusion criteria. After several selections and assessments, the basic information of the included trails (first author, title, and number of each treatment), patient characteristics (age, sex, ethnicity and race, duration of untreated attacks, historical and treated-attack coma, and most bothersome symptom), and outcome measures were used to extract the data (**Table 1**).

**Table 1:** Characteristics of the Included Studies and Outcome Events.

Trials	Marcus et al., 2014 (NCT1430442)	Croop et al., 2019 (NCT3461757)	Lipton et al., 2019 (NCT03237845)	Lipton, 2018 (NCT03235479)
**1. Information of the Included Trials**				
***Regions***	3 centers in USA	4 centers in UK and USA	4 centers in USA	4 centers in USA
***Phases***	III	III	IIB/III	IIB/III
***Publication***	Cephalalgia	Lancet Neurology	New England Journal of Medicine	Headache
**2. Eligibility Criteria and Study Design**				
***Inclusion Criteria***	Acute migraine	Acute migraine	Acute migraine	Acute migraine
	Age:18-65 years old	Age>18 years old	Age>18 years old	Age>18 years old
	At least one-year history of migraine	At least one-year history of migraine	At least one-year history of migraine	At least one-year history of migraine
	Two to seven attacks in each 3 months	At least two attacks in each month	Two to eight attacks in each month	Two to eight attacks in each month
***Exclusion Criteria***	History of basilar-type migraine	History of serious illness	History of any clinically significant or unstable medical condition	History of any clinically significant or unstable medical condition
	History of stroke/transient ischemic attacks	Alcohol or drug abuse	Alcohol or drug abuse and substance-use disorder	Alcohol or drug abuse and substance-use disorder
***Study Design***	Rimegepant 10mg, 25mg, 75mg, 150mg, 300mg, 600mg or Sumatriptan 100mg or placebo	Rimegepant 75mg or placebo	Rimegepant 75mg or placebo	Rimegepant 75mg or placebo
**3. Outcomes Assessments**				
***Primary outcomes***	Freedom from pain at 2h postdose	Freedom from pain at 2h postdose	Freedom from pain at 2h postdose	Freedom from pain at 2h postdose
	Freedom from most bothersome pain at 2h postdose	Freedom from most bothersome pain at 2h postdose	Freedom from most bothersome pain at 2h postdose	Freedom from most bothersome pain at 2h postdose
***Safety outcomes***	Nausea, Dizziness, Vomiting, Diarrhea, Paresthesia, Dysgeusia, Chest discomfort, Myalgia	Nausea, Urinary tract infection, Dizziness, Adverse events related to treatment	Nausea, Urinary tract infection, serious adverse events, liver-function	Nausea, Urinary tract infection, serious adverse events, liver-function

### Outcome Measures

The primary outcomes included freedom from pain at 2 hr post dose, freedom from most bothersome symptom at 2 hr post dose, and pain relief at 2 hr post dose. Secondary endpoints included freedom from photophobia at 2 hr post dose, freedom from phonophobia at 2 hr post dose, freedom from nausea at 2 hr post dose, sustained pain relief from 2 to 24 hr post dose, sustained freedom from pain from 2 to 24 hr post dose, sustained pain relief from 2 to 48 hr post dose, and sustained freedom from pain from 2 to 48 hr postdose. The adverse events included nausea, urinary tract infection, dizziness, and serum AST or ALT above ULN.

### Summary Measures and Synthesis of Results

STATA (Version 12.0) was used to evaluate the data. Estimated proportions with the risk ratio (relative risk [RR]; 95% confidence interval [CI]) were calculated using a random-effects model. Statistical heterogeneity was estimated by the *I* ^2^ statistic: *I* ^2^ < 30% represents “low heterogeneity”, 30% < *I* ^2^ < 50% means “moderate heterogeneity” and *I* ^2^ > 50% means “substantial heterogeneity”. The stability of the consolidated results was explored by sensitivity analysis. A value less than 0.05 P was considered to be significant for all analyses and the results were made up of two-tailed tests.

### Risk of Bias

The Review manager 5.2 software was used to create the risk of bias plot in individual studies. The Cochrane collaboration uniform criteria were used for assessing the risk of bias of RCTs. Selection bias, performance bias, detection bias, attrition bias, reporting bias, and other possible biases were included in the criteria.

## Results

### Search Results

**Figure 1:**
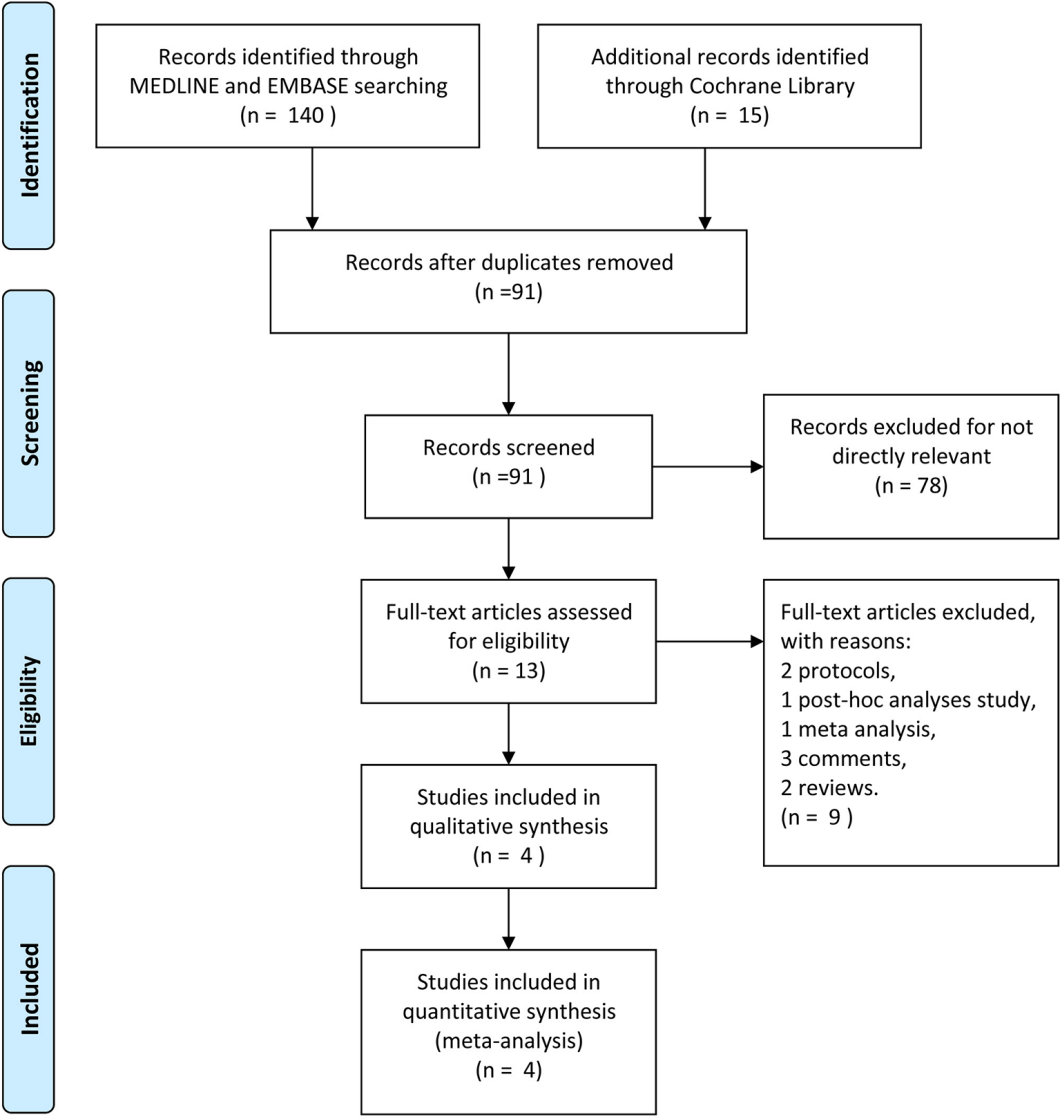
The study search, selection, and inclusion process.

A total of 155 researches and abstracts from Pubmed, Embase, and Cochrane library were identified. Sixty-four studies were removed due to duplicates. Seventy-eight studies were removed because they were irrelevant, such as research on other drugs or into the etiological analysis of migraine, etc. After removing duplicates and uncorrelated titles, 13 of these articles were directly related to the topic of interest. Among them, nine full-text articles were excluded, which included two protocols, one post-hoc analysis study, one network meta-analysis, three comments, and two reviews. Finally, four RCTs containing 3,827 patients were included in our meta-analysis. The specific process and included study characteristics are shown in **Figure 1**.

### Assessment of Primary Outcomes

**Figure 2:**
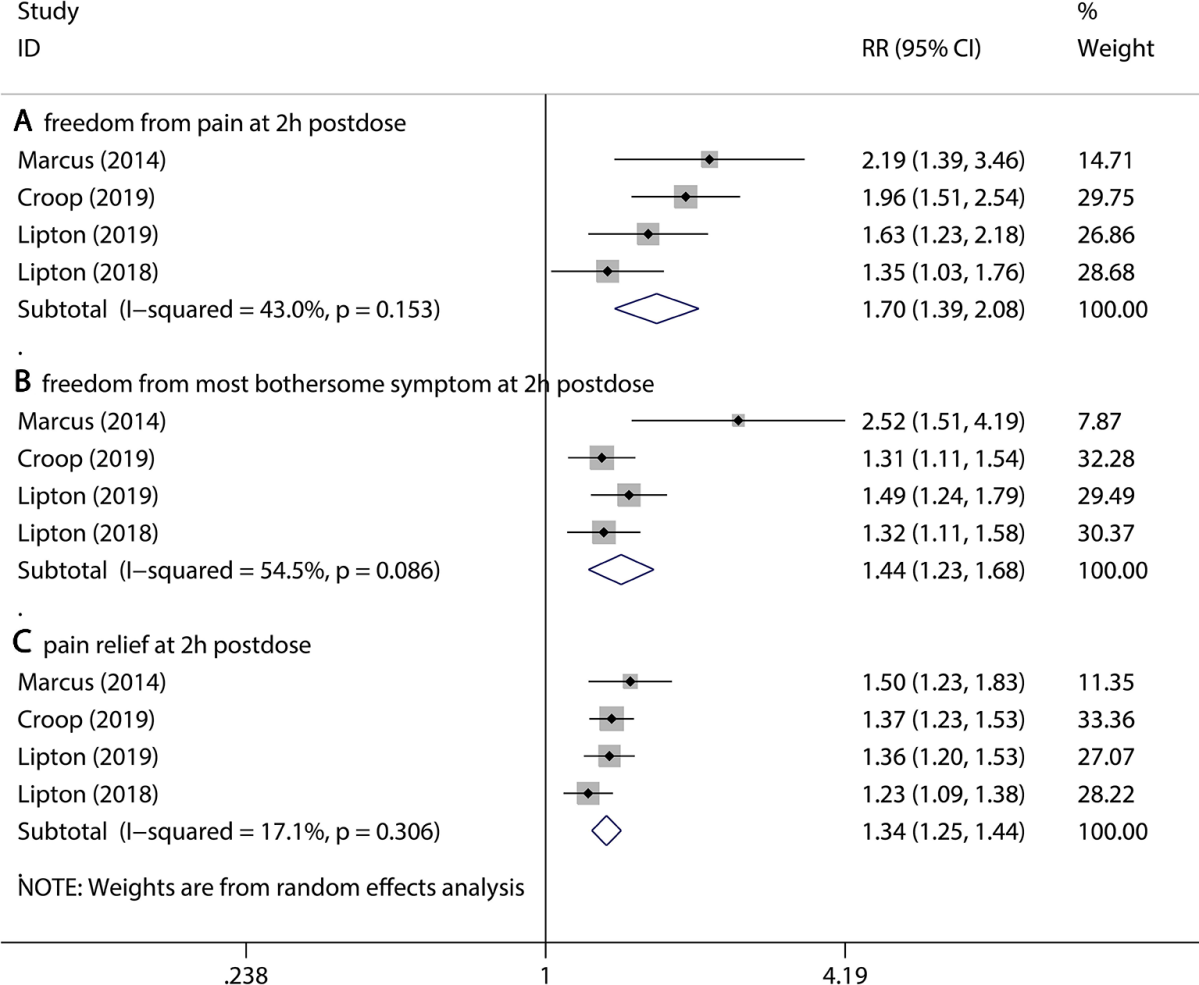
The pooled RR of primary outcomes. Notes: The black diamond indicates the estimated RR for each RCT. The gray box around each diamond indicates the estimated weight of each RCT, and the extending lines indicate the estimated 95% CI of RR for each RCT. The diamond indicates the estimated RR (95% CI) for all patients together. **(A)** Freedom from pain at 2 hr post dose. **(B)** Freedom from most bothersome symptom at 2 hr post dose. **(C)** Pain relief at 2 hr post dose. Weights are from a random-effects analysis. CI, confidence interval; RCT, randomized controlled trial; RR, relative risk.

The primary outcomes include freedom from pain 2 hr post dose, freedom from most bothersome symptoms 2 hr post dose, and pain relief 2 hr post dose. Treatment with 75 mg rimegepant showed significant efficacy compared to the placebo with respect to all of the primary outcomes (freedom from pain 2 hr post dose, 20.6% vs 12.5% for rimegepant vs placebo RR = 1.70, 95% CI:1.39–2.08, P < 0.001; freedom from most bothersome symptoms, 36.0% vs 25.1% for rimegepant vs placebo RR = 1.44, 95% CI:1.23–1.68, P < 0.001; pain relief 2hr post dose, 58.6% vs 44.6% for rimegepant vs placebo RR = 1.34, 95% CI:1.25–1.44, P < 0.001, **Figure 2**). The *I* ^2^ regarding the result of freedom from most bothersome symptoms was over 50%. Therefore, a sensitivity analysis was performed to detect the statistical heterogeneity. The sensitivity analysis confirmed that the results were stable (**Supplement I**).

### Assessment of Secondary Outcomes

We evaluated the improvement of the accompanying symptoms in the short term, and we also looked at whether the improvement of symptoms will be sustainable.

**Figure 3:**
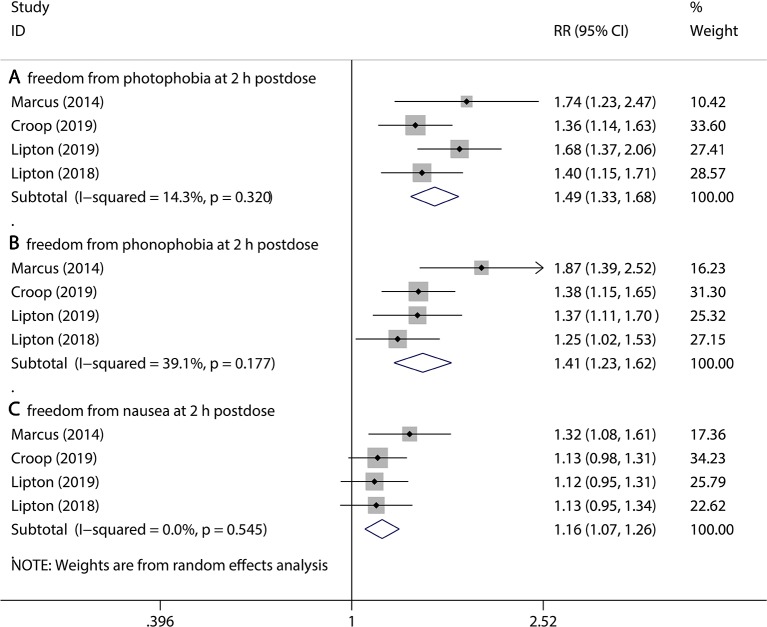
The pooled RR of secondary outcomes (accompanying symptoms). Notes: The black diamond indicates the estimated RR for each RCT. The gray box around each diamond indicates the estimated weight of each RCT, and the extending lines indicate the estimated 95% CI of RR for each RCT. The diamond indicates the estimated RR (95% CI) for all patients together. **(A)** Freedom from photophobia at 2 hr post dose. **(B)** Freedom from phonophobia at 2 hr post dose. **(C)** Freedom from nausea at 2 hr post dose. Weights are from a random-effects analysis. CI, confidence interval; RCT, randomized controlled trial; RR, relative risk.

**Figure 4:**
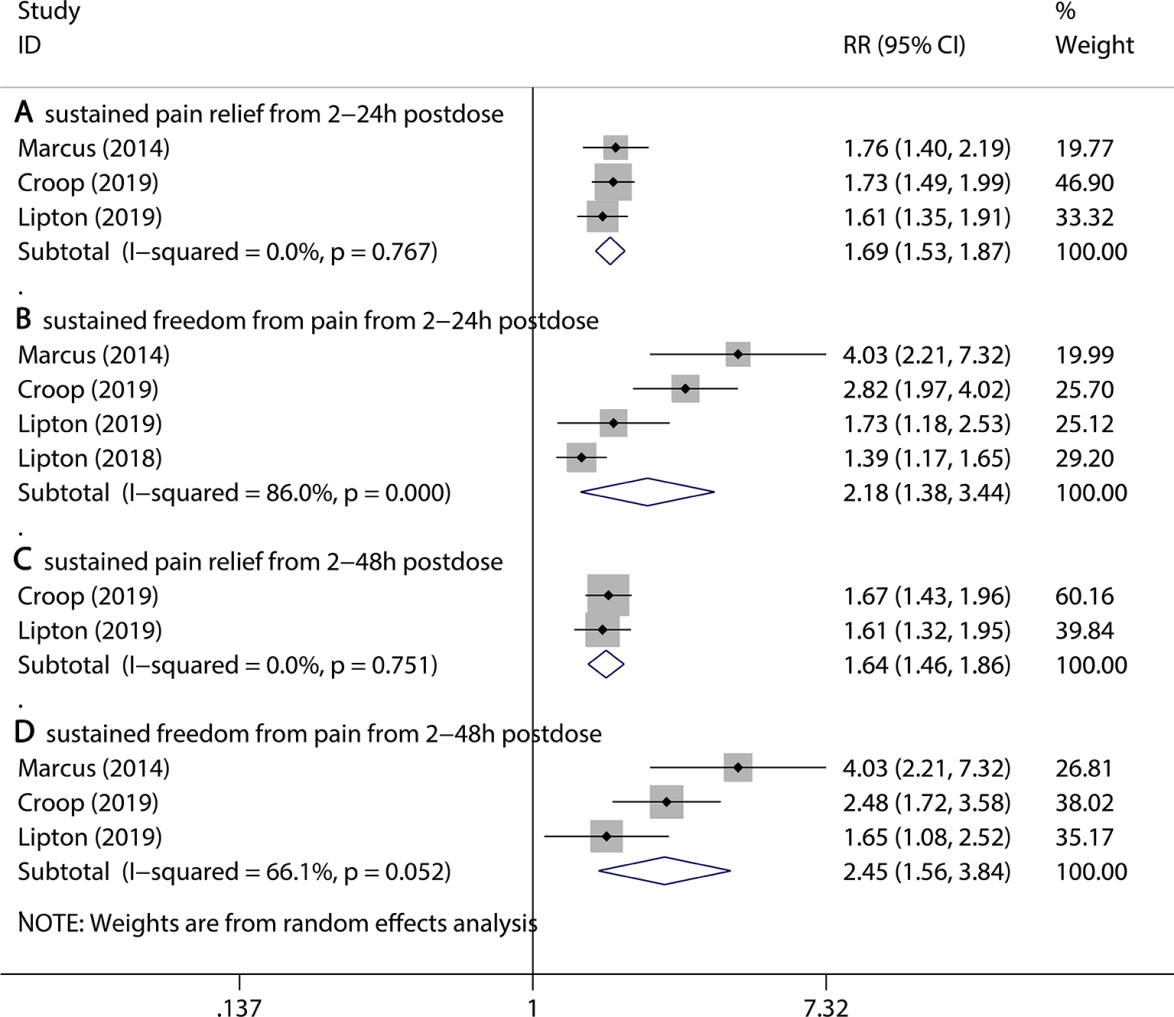
The pooled RR of secondary outcomes (sustained symptoms). Notes: The black diamond indicates the estimated RR for each RCT. The gray box around each diamond indicates the estimated weight of each RCT, and the extending lines indicate the estimated 95% CI of RR for each RCT. The diamond indicates the estimated RR (95% CI) for all patients together. **(A)** Sustained pain relief from 2 to 24 hr post dose. **(B)** Sustained freedom from pain from 2 to 24 hr post dose. **(C)** Sustained pain relief from 2 to 48 hr post dose. **(D)** Sustained freedom from pain from 2 to 48 hr post dose. Weights are from a random-effects analysis. CI, confidence interval; RCT, randomized controlled trial; RR, relative risk.

Firstly, three kinds of outcomes were assessed, including freedom from photophobia at 2 hr post dose, freedom from phonophobia at 2 hr post dose, and freedom from nausea at 2 hr post dose. Compared to the placebo group, the 75 mg rimegepant group had a significant reduction in accompanying symptoms (freedom from photophobia at 2 hr post dose, 35.5% vs 23.9% for rimegepant vs placebo RR = 1.49, 95% CI:1.33–1.68, P < 0.001; freedom from phonophobia at 2 hr post dose, 40.1% vs 29.1% for rimegepant vs placebo RR = 1.41, 95% CI:1.23–1.62, P < 0.001; freedom from nausea at 2 hr post dose, 50.3% vs 44.7% for rimegepant vs placebo RR = 1.16, 95% CI:1.07–1.26, P = 0.001, **Figure 3**). Secondly, two periods of sustained improvement covering four kinds of outcomes were assessed, including sustained pain relief from 2 to 24 hr postdose, sustained freedom from pain from 2 to 24 hr postdose, sustained pain relief from 2 to 48 hr postdose, and sustained freedom from pain from 2 to 48 hr postdose. All the four kinds of outcomes are better in the rimegepant group than the placebo group (in sustained pain relief from 2 to 24 hr pos tdose, 47.1% vs 29.4% for rimegepant vs placebo RR = 1.69, 95% CI = 1.53–1.87, P < 0.001; in sustained freedom from pain from 2 to 24 hr post dose, 22.1% vs 12.3% for rimegepant vs placebo RR = 2.18, 95% CI = 1.38–3.44, P = 0.001; in sustained pain relief from 2 to 48 hr post dose, 39.6% vs 24.1% for rimegepant vs placebo RR = 1.64, 95% CI = 1.46–1.86, P < 0.001; in sustained freedom from pain from 2 to 48 hr post dose, 12.9% vs 5.9% for rimegepant vs placebo RR = 2.45, 95% CI = 1.56–3.84, P < 0.001) (**Figure 4**). The *I* ^2^ regarding the result of sustained freedom from pain from 2 to 24 hr postdose was over 50%. Therefore, a sensitivity analysis was performed to detect the statistical heterogeneity. The sensitivity analysis confirmed that one trail was highly sensitive (**Supplement II**). After excluding a highly sensitive trial, the result of sustained freedom from pain from 2 to 24 hr post dose had no change (**Supplement III**). The combination result showed the use of rimegepant produced a significant improvement in sustained freedom from pain from 2 to 24hr post dose compared with placebo (15.1% vs 6.4% for rimegepant vs placebo RR = 2.59, 95% CI = 1.66–4.06, P < 0.001). The *I* ^2^ regarding the result of sustained freedom from pain from 2 to 48 hr post dose is over 50%. Therefore, sensitivity analysis was performed to detect the statistical heterogeneity. The sensitivity analysis confirmed that the results were stable (**Supplement IV**).

### Assessment of Adverse Events

**Figure 5:**
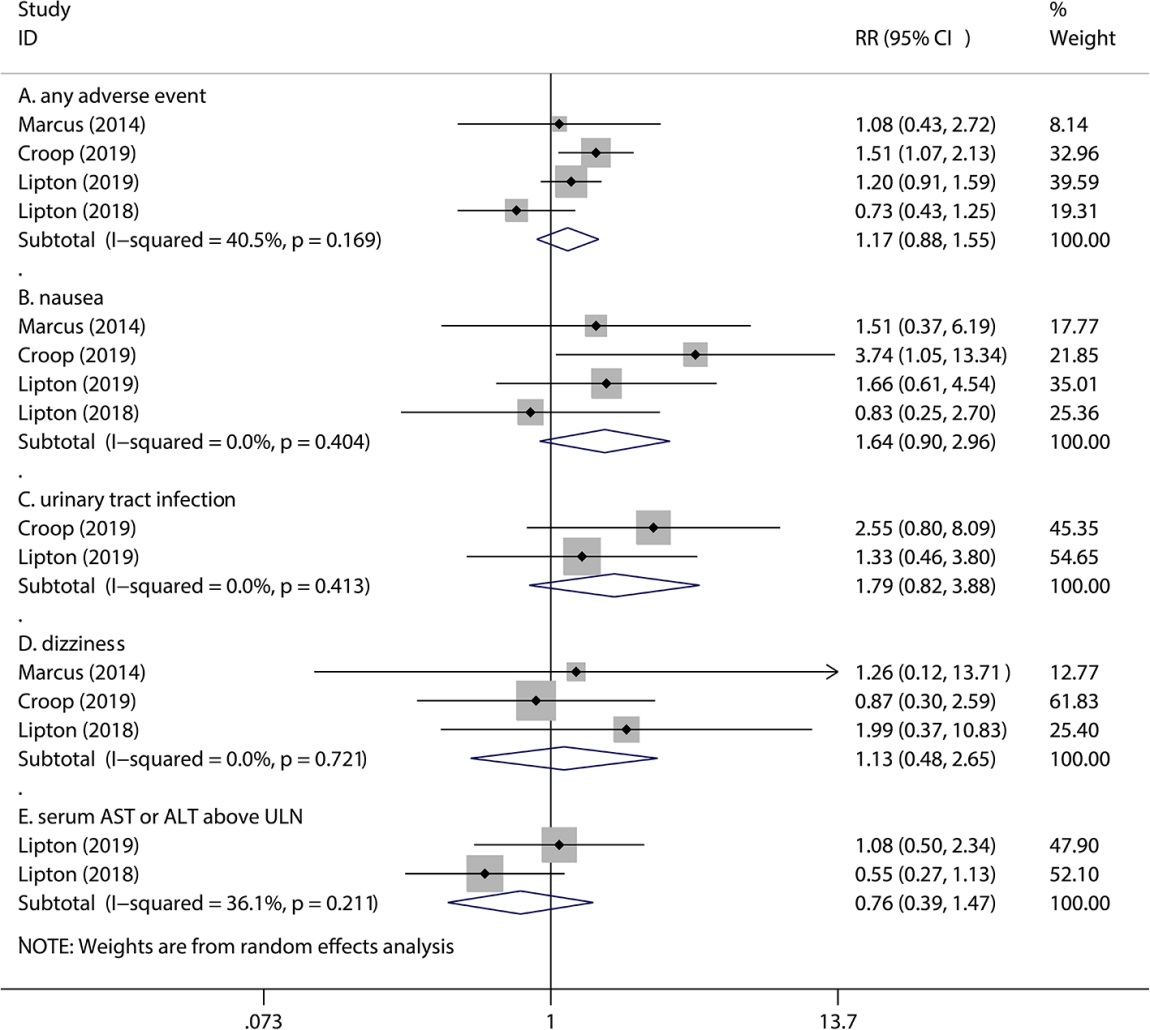
The pooled RR of adverse events. Notes: The black diamond indicates the estimated RR for each RCT. The gray box around each diamond indicates the estimated weight of each RCT, and the extending lines indicate the estimated 95% CI of RR for each RCT. The diamond indicates the estimated RR (95% CI) for all patients together. **(A)** Any adverse event. **(B)** Nausea. **(C)** Urinary tract infection. **(D)** Dizziness. **(E)** Serum AST or ALT above ULN. Weights are from a random-effects analysis. Abbreviations: CI, confidence interval; RCT, randomized controlled trial; RR, relative risk.

In recent studies, four kinds of adverse events are most common in rimegepant treatment of migraine. As a matter of fact, the adverse events of 75 mg rimegepant treatment of migraine were similar to those of the placebo. These safety outcomes included nausea (1.6% vs 1.0% for rimegepant vs placebo RR = 1.64, 95% CI = 0.90–2.96,P = 0.105), urinary tract infection (1.5% vs 0.8% for rimegepant vs placebo RR = 1.79, 95% CI = 0.82–3.88, P = 0.144), dizziness (0.8% vs 0.8% for rimegepant vs placebo RR = 1.13, 95% CI = 0.48–2.65, P = 0.781), and serum aspartate aminotransferase (AST) or alanine aminotransferase (ALT) above the upper limit of normal (ULN) (2.2% vs 2.9% for rimegepant vs placebo RR = 0.76, 95% CI = 0.39–1.47, P = 0.414). A total of 75 mg rimegepant showed a neutral effect on any adverse events compared with placebo (4.4% vs 3.7% for rimegepant vs placebo RR = 1.17, 95% CI = 0.88–1.55, P = 0.284) (**Figure 5**).

### Risk of Bias in Included Studies

**Figure 6:**
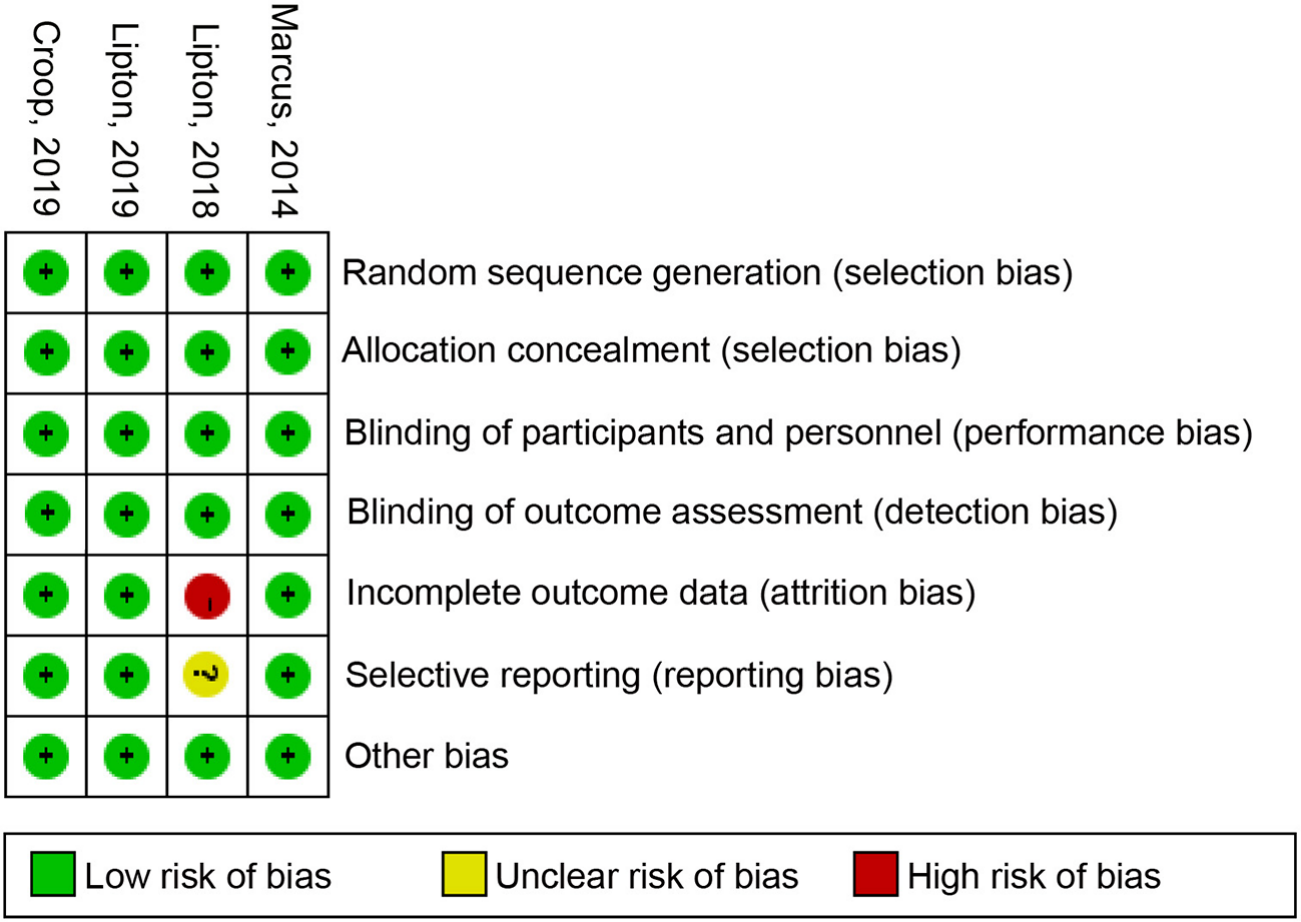
Risk of bias: a summary table for each risk of bias item for each study.

The independent risk of bias of the four included studies are shown in **Figure 6** in detail. The risk for incomplete outcome data bias is high in the Lipton study (2018). For selective reporting, the Lipton study had an unclear risk of bias. In addition to these two measures, other studies had low risks of bias.

## Discussion

In our meta-analysis, we pooled 3,827 patients from four multicentered RCTs, which provided higher levels of clinical reliability for evaluating the efficacy and safety of the rimegepant. Based on the results of this meta-analysis, rimegepant was significantly more effective compared to placebo measured by the improvement of symptoms in the acute phase of migraine. By comparing three primary outcomes, whether in the freedom or relief from pain within 2 hours, or the freedom from the most bothersome symptoms within 2 hours, rimegepant had the obvious effect of relieving symptoms. After using rimegepant, the duration of pain freedom or relief after using the rimegepant was significantly longer than that after using placebo; in addition, the symptoms of photophobia or phonophobia were more obviously improved than nausea. This may prove that rimegepant is more effective for patients with the symptom of photophobia or phonophobia. After the meta-analysis, it was found that there was no difference in adverse events between the rimegepant and the placebo.

Previously, triptans were the most frequently used drugs for acute management of migraine, but 10% of the patients who received triptans developed cardiovascular symptoms, and, due to their potent vasoconstrict effect, triptans were contraindicated for patients with cardiovascular diseases. As a CGRP blocker, rimegepant has good efficacy and safety. (Edvinsson, 2019) Previous network meta-analysis (NMA) compared all six kinds of CGRP blockers with triptans and placebo and showed that olcegepant was the most effective and toxic of them and that rimegepant had moderate efficacy and moderate toxicity (Xu and Sun, 2019). In this NMA, it did not describe the specific values of various gepants, but it gave an order of efficacy: olcegepant, BI-44370, ubrogepant, rimegepant, telcagepant, and MK-3207. Although the olcegepant had the best efficacy, it was administered intravenously while other drugs were administrated orally. This NMA also indicated that the rimegepant had more toxicity than the placebo and may have caused many adverse events. However, the study only pooled 547 patients for rimegepant group. Therefore, our meta-analysis added three RCTs, including 3,280 patients, to further assess the efficacy and safety of rimegepant.

Due to the belief that drug treatment of acute migraine is aimed mainly at the short term, previous studies have focused on freedom from pain 2 hours post dose and freedom from the most bothersome symptoms (such as photophobia, phobia, nausea, etc.) 2 hours post dose. This meta-analysis also included pain relief 2 hours post dose as a primary outcome to draw a comparison with the improvement of symptoms in patients with different degrees of pain. For patients with migraine, symptoms can be divided into mild, moderate, and severe pain. Severe pain may be more difficult to relieve than mild or moderate pain. If freedom from pain only is considered as the main measure of outcome of treatment, it will inevitably lead to deviations. Therefore, we also use pain relief as a primary outcome. For these three prognoses (freedom or relief from pain within 2 hours and freedom from the most bothersome symptoms within 2 hours), rimegepant was 8.1%, 14.0%, and 11.9% more effective than the placebo, respectively. This indicated that the effect of rimegepant on pain relief was more obvious than its effect on freedom from pain or the most bothersome symptoms.

These patients, due to the diversity of bothersome symptoms, can be roughly divided into three most common symptoms: photophobia, phonophobia, and nausea. However, in terms of improvement of nausea symptoms, the conclusions of the four RCTs were not consistent. After meta-analysis, it was proven that rimegepant had a significant effect on the improvement of nausea, and for these three prognoses, the rimegepant was 11.5%, 11.0%, and 5.6% more effective than the placebo, respectively. After using rimegepant, the symptoms of photophobia or phonophobia were more obviously improved than nausea. Meanwhile, in both Lipton studies, the most bothersome symptom for patients was photophobia. (Croop et al., 2019; Lipton et al., 2019) This thus exemplifies the effectiveness of rimegepant in the acute treatment of migraine.

After 24 hours of rimegepant, patients had 9.8% more sustained pain freedom and 17.8% more sustained pain relief than with the placebo. After 48 hours, 7.0% people had no pain and 15.5% people still experienced sustained pain relief as compared to the placebo. By comparison, it can be seen that the use of rimegepant did not only relieve the migraine in the acute phase, but it also stabilized the symptoms of migraine to a certain extent, ensuring that the migraine did not recur in the short term. Also, the percentage of people who experienced sustained pain relief was greater than those that experienced sustained freedom from pain. It was indicated that rimegepant is more effective for pain relief. In addition, it can be seen that there was not much difference in the symptoms in the 24-hour to 48-hour interval, which may be related to the rate of metabolism of the rimegepant in the body.

To investigate the safety of rimegepant, we selected several of the most common adverse events of different doses in Marcus’s study for evaluation. Although the results of the rimegepant safety assessment were different in the four RCTs, after meta-analysis, we found that there was no difference between the rimegepant and placebo (4.4% vs 3.7%). Previous studies of other types of CGRP blockers, such as olcegepant, have found that the most obvious adverse event of CGRP blockers is damage to liver function. Two of the four RCTs used liver function damage as a criterion for assessing the safety of rimegepant. In one of the Lipton studies, the use of rimegepant could cause liver injury, but in the other study there was no difference between the rimegepant and placebo. Meta-analysis showed there was no significant liver damage found in the rimegepant compared with the placebo (2.2% vs 2.9%).

The purpose of developing this new drug is to avoid the adverse events of using other drugs to treat migraine. For example, triptans can cause adverse cardiovascular events, and non-steroidal anti-inflammatory drugs can cause digestive ulcers. Compared with the current mainstay of specific acute migraine treatment, CGRP receptor antagonists did not cause vasoconstriction and could, instead, decrease the likelihood of adverse cardiovascular events. (Verheggen et al., 2002; Petersen et al., 2005; Lynch et al., 2009; Chan et al., 2010) Although the sites of action of gepants and triptans are different, considering the adverse events of both drugs, a combination of the two is not feasible because of the high risk for developing adverse events. These four RCTs were not mentioned in the context of adverse cardiovascular events. In the previous NMA, the olcegepant, BI-44370, and triptans had better efficacy than rimegepant. However, these three drugs also exhibited significant adverse events. Also, in the previous NMA, it was indicated that the rimegepant had more toxicity than the placebo. Our meta-analysis showed that rimegepant was safe. Therefore, rimegepant has more of an advantage in terms of safety than other CGRP blockers or triptans. However, there is still a limitation that rare and serious adverse events may exist that were not identified during the four RCTs. Our conclusion is limited to the more common adverse events, and further research is needed to prove whether rimegepant cause more serious adverse events.

### Limitation

A limitation of this meta-analysis is that it can prove that rimegepant is effective for migraine, but it cannot prove that rimegepant is superior in effectiveness to other kinds of drugs. In 2014, Marcus’s study (Marcus et al., 2014) found that the 75 mg of rimegepant could produce a similar effect to higher doses of rimegepant and 100 mg of sumatriptan with fewer adverse events than 100 mg of sumatriptan. Therefore, in the other three studies, 75 mg of rimegepant was chosen as a comparison to the placebo. However, there were large variations in the doses selected by the Ronald study, which used 25 mg, 75 mg, 150 mg, 300 mg, and 600 mg. In this study, 75 mg of rimegepant has a slightly worse effect than 100 mg of sumatriptan, and a more reliable conclusion might be obtained if other doses (50 mg, 100 mg, or 125 mg) of rimegepant were used in comparison with the 100 mg of sumatriptan. What’s more, on an individual level, it is possible that some patients may benefit from a higher dose. This also needs further research. In the other three RCTs, there was no comparison between the efficacy of rimegepant and sumatriptan. Moreover, there was no comparison of the effectiveness of rimegepant with other CGRP blockers. Therefore, there is still a need for more studies.

The assessment of pain was subjective to the patients, and relevant outcome scales were not used in previous RCTs. All the patients were followed up by using an electronic diary to log their current pain and most bothersome migraine-associated symptoms. There may be a bias in patients’ assessment of their current symptoms.

Another limitation, regarding to selection of patients, is that subjects of these four RCTs were roughly 40-year-old non-Hispanic and non-Latino white women with a BMI of about 31. Since the original data of each institute could not be obtained, the study was limited to the abovementioned characteristics of the population, and there was no subgroup analysis of populations of other ages, statures, races, and genders. In this regard, the people with the above characteristics had the highest incidence of migraine, so the effectiveness of the rimegepant is still meaningful. In addition, in order to prove that the scope of application of rimegepant can be more extensive, there should be more research done on migraine patients of different characteristics.

## Conclusion

Rimegepant exhibits good efficacy and safety for the acute treatment of migraine. A dose of 75 mg rimegepant was proven to be effective against acute migraine headache as measured by freedom from pain and bothersome symptoms or pain relief 2 hours post dose after drug ingestion as compared to the placebo. The use of 75 mg rimegepant was not related to a significant increase in these specific adverse events.

## Author Contributions

ZhW was the principal investigator. BG and YY designed the study and developed the analysis plan. ZiW and YS analyzed the data and performed the meta-analysis. BG, YY, and ZC contributed to the writing of the article. YZ and ZhW revised the manuscript and polished the language.

## Funding

This work was supported by the Suzhou Health Talents Training Project (GSWS2019002).

## Conflict of Interest

The authors declare that the research was conducted in the absence of any commercial or financial relationships that could be construed as a potential conflict of interest.

## Supplementary Material

The Supplementary Material for this article can be found online at: https://www.frontiersin.org/articles/10.3389/fphar.2019.01577/full#supplementary-material
