# Supplementary material for: Efficacy and Safety of Rimegepant for the Acute Treatment of Migraine: Evidence From Randomized Controlled Trials
Source: Front Pharmacol. 2020 Jan 24;10:1577. doi: 10.3389/fphar.2019.01577 (PMC6992660; doi:10.3389/fphar.2019.01577)

**Title:** **Efficacy and safety of rimegepant for the acute treatment of migraine: evidence from randomized controlled trials.**

**Bixi Gao^1#^, Yanbo Yang^1,#^, Zilan Wang^1^, Yue Sun^1^, Zhouqing Chen^1^, Yun Zhu^1,*^, Zhong Wang ^1,*^**

**^1^** ***Department of Neurosurgery & Brain and Nerve Research Laboratory,*** ***The First Affiliated Hospital of Soochow University, Suzhou, Jiangsu Province, 215006, China***

**Supplement I**

**Sensitivity analysis of overall survival showed that all of the consolidated results were stable.**

**
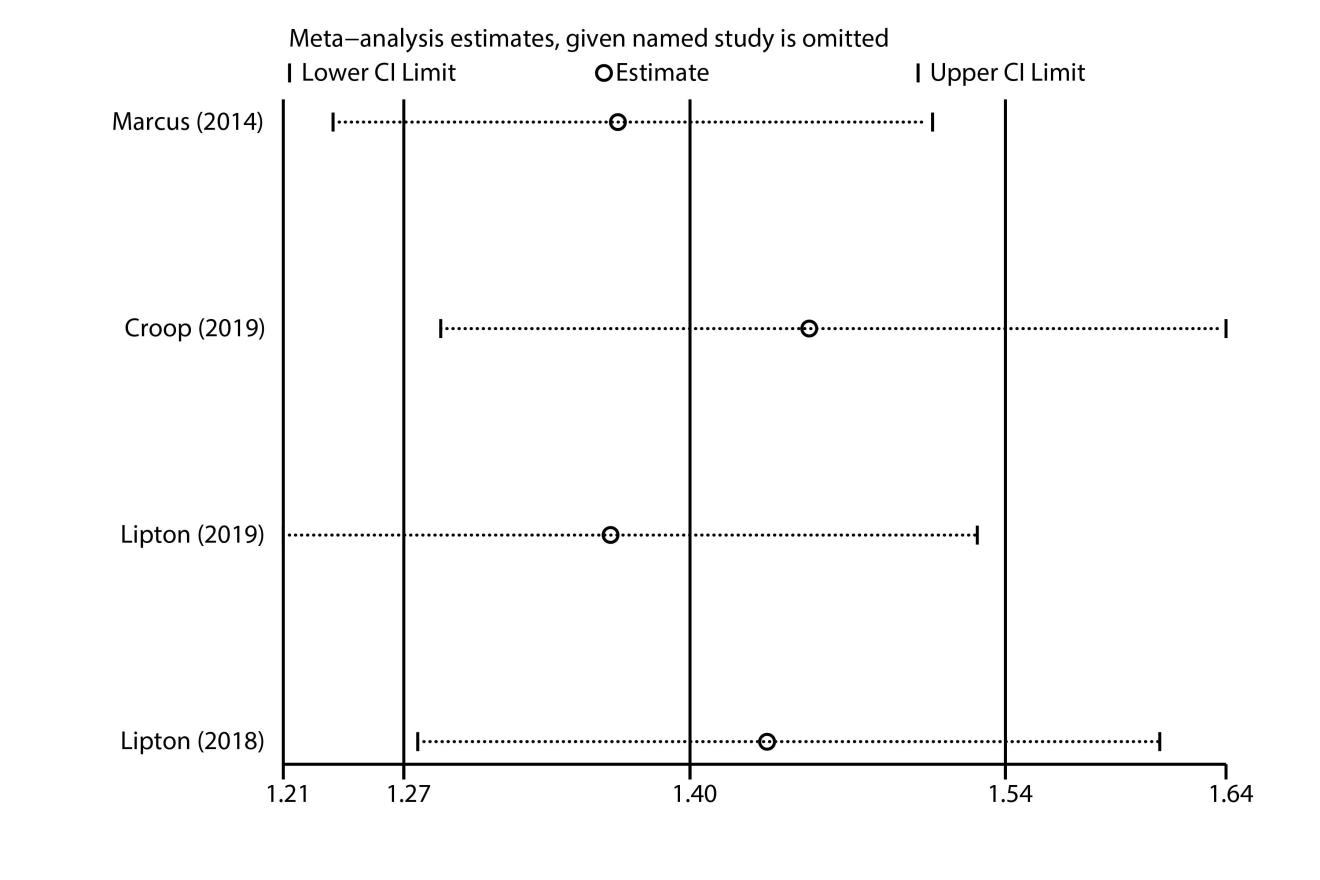
**

**Supplement II**

**Sustained freedom from pain from 2-24hr postdose sensitivity analysis showed that fourth trial (****Lipton, 2018) was highly sensitive and the remaining RCTs were within the confidence interval.**

**
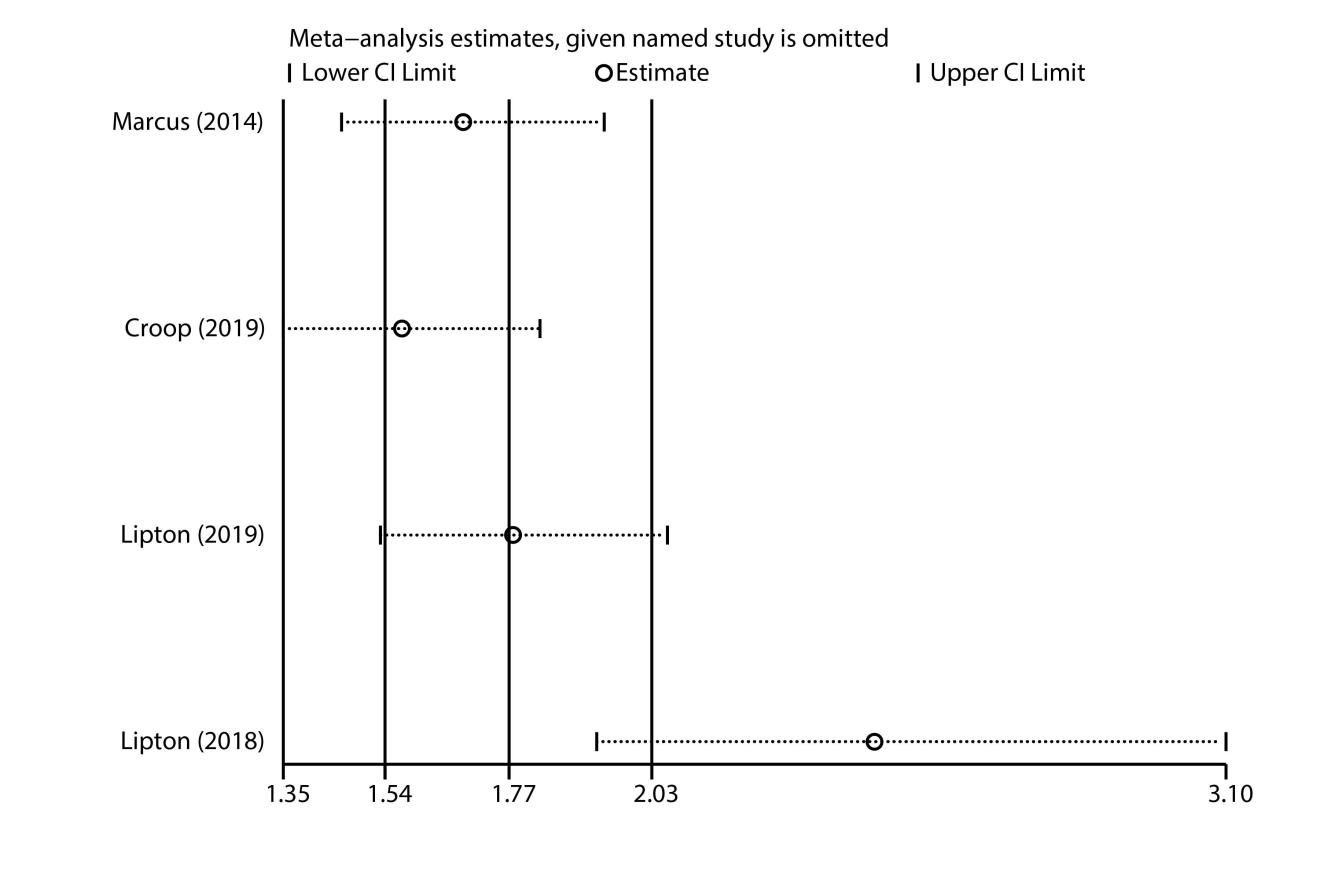
**

**Supplement III**

**The pooled** **hazard ratio of the sustained freedom from pain from 2-24hr postdose outcomes after exclusion highly sensitive trial (Lipton, 2018). The diamond indicates the estimated hazard ratio (95% confidence interval) for all patients together.**

**
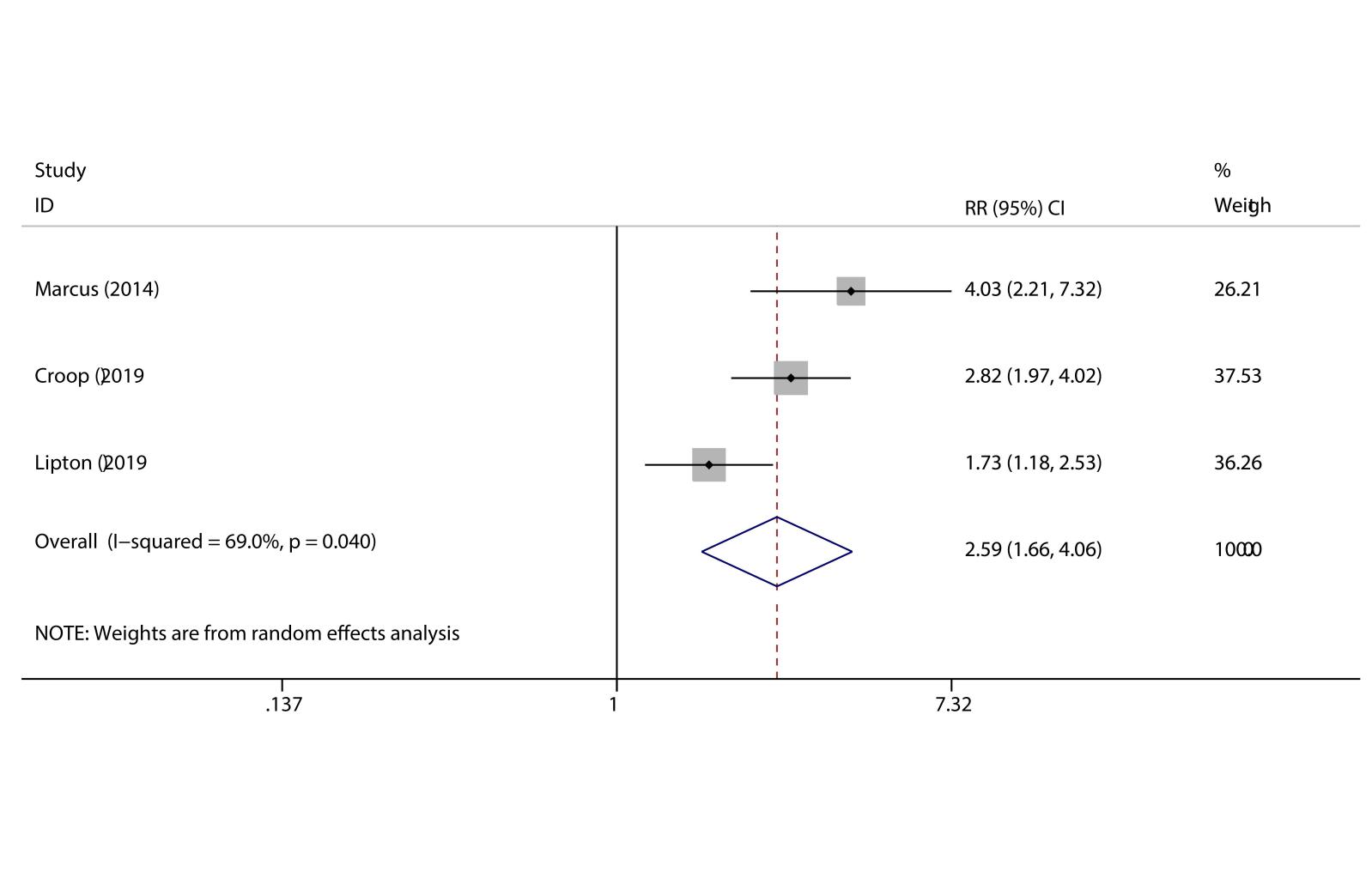
**

**Supplement IV**

**Sensitivity analysis of overall survival showed that all of the consolidated results were stable.**


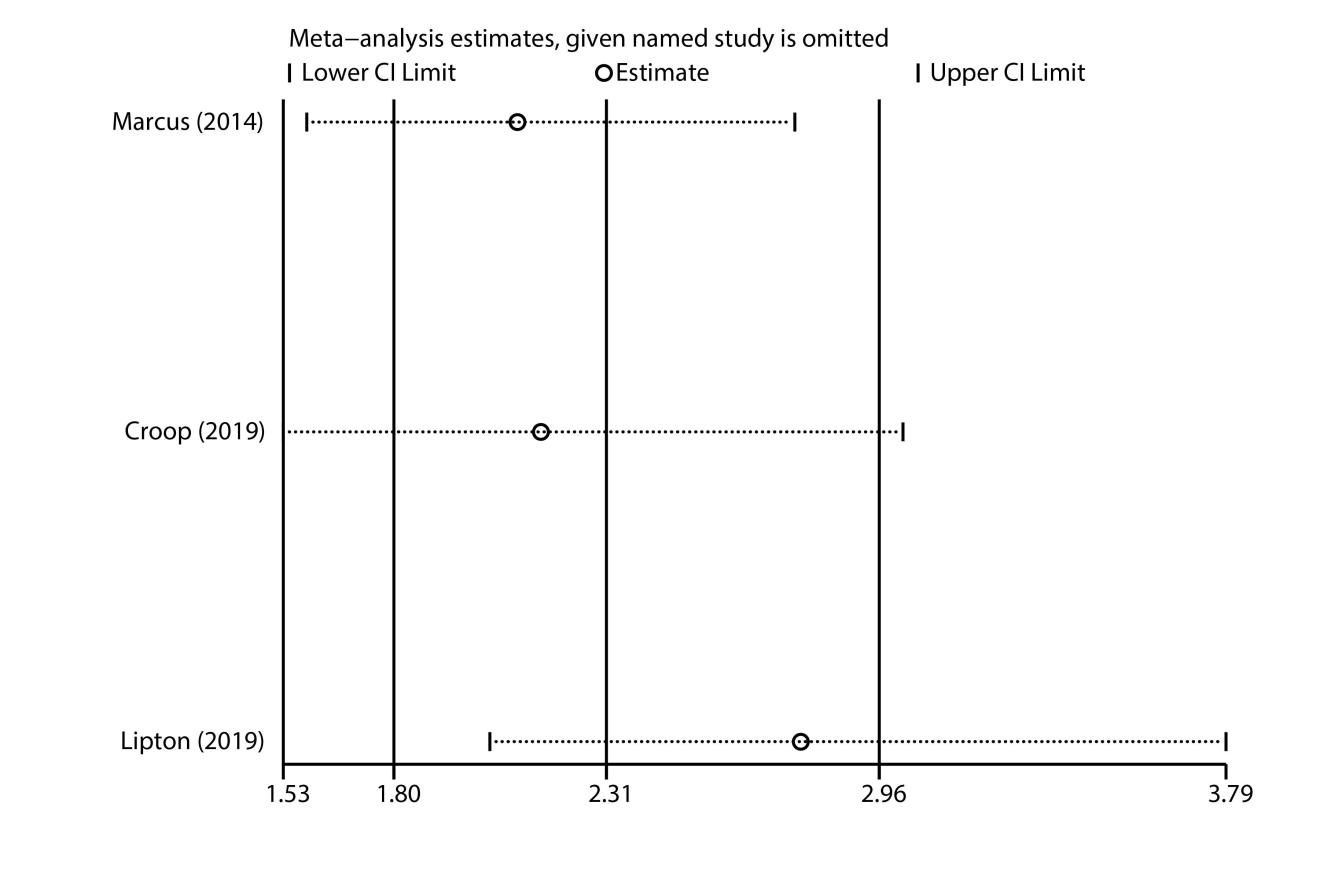

Supplement: Supplementary file 1 [file DataSheet_1.docx]
