# Supplementary material for: Efficacy and Safety of Rimegepant for the Acute Treatment of Migraine: Evidence From Randomized Controlled Trials
Source: Front Pharmacol. 2020 Jan 24;10:1577. doi: 10.3389/fphar.2019.01577 (PMC6992660; doi:10.3389/fphar.2019.01577)
Supplement: Supplementary file 2 [file Table_1.docx]

**Table 1: Characteristics of the Included Studies and Outcome Events**

| **Trials** | **Marcus, 2014**  **(****NCT1430442)** | **Croop, 2019**  **(NCT3461757)** | **Lipton, 2019**  **(NCT03237845)** | **Lipton, 2018**  **(NCT03235479)** |
| --- | --- | --- | --- | --- |
| 1. **Information of the Included Trials** | | | | |
| ***Regions*** | 3 centers in USA | 4 centers in UK and USA | 4 centers in USA | 4 centers in USA |
| ***Phases*** | III | III | IIB/III | IIB/III |
| ***Publication*** | Cephalalgia | Lancet Neurology | New England Journal of Medicine | Headache |
| 1. **Eligibility Criteria and Study Design** | | | | |
| ***Inclusion Criteria*** | Acute migraine  Age:18-65 years old  At least one-year history of migraine  Two to seven attacks in each 3 months | Acute migraine  Age>18 years old  At least one-year history of migraine  At least two attacks in each month | Acute migraine  Age>18 years old  At least one-year history of migraine  Two to eight attacks in each month | Acute migraine  Age>18 years old  At least one-year history of migraine  Two to eight attacks in each month |
| ***Exclusion Criteria*** | History of basilar-type migraine  History of stroke/transient ischemic attacks | History of serious illness  Alcohol or drug abuse | History of any clinically significant or unstable medical condition  Alcohol or drug abuse and substance-use disorder | History of any clinically significant or unstable medical condition  Alcohol or drug abuse and substance-use disorder |
| ***Study Design*** | Rimegepant 10mg, 25mg, 75mg, 150mg, 300mg, 600mg or Sumatriptan 100mg or placebo | Rimegepant 75mg or placebo | Rimegepant 75mg or placebo | Rimegepant 75mg or placebo |
| 1. **Outcomes Assessments** | | | | |
| ***Primary outcomes*** | Freedom from pain at 2h postdose  Freedom from most bothersome pain at 2h postdose | Freedom from pain at 2h postdose  Freedom from most bothersome pain at 2h postdose | Freedom from pain at 2h postdose  Freedom from most bothersome pain at 2h postdose | Freedom from pain at 2h postdose  Freedom from most bothersome pain at 2h postdose |
| ***Safety outcomes*** | Nausea, Dizziness, Vomiting, Diarrhea, Paresthesia, Dysgeusia, Chest discomfort, Myalgia | Nausea, Urinary tract infection, Dizziness, Adverse events related to treatment | Nausea, Urinary tract infection, serious adverse events, liver-function | Nausea, Urinary tract infection, serious adverse events, liver-function |
